# Transcatheter Aortic Valve Implantation/Replacement (TAVI/TAVR): How It Started, How It’s Going, and Where It’s Going

**DOI:** 10.3390/jcm15135242

**Published:** 2026-07-04

**Authors:** Alok Shah, Amr Gamal, Hesham Abdelaziz, Matthew Luckie, Andrew Wiper, Ranjit More, Tawfiq Choudhury

**Affiliations:** 1 Department of Cardiology, Sir HN Reliance Foundation Hospital and Research Centre, Mumbai 400004, Maharashtra, India; 2 Lancashire Cardiac Centre, Blackpool Teaching Hospitals NHS Foundation Trust, Blackpool, Lancashire FY3 8NR, UK

**Keywords:** TAVI, transcatheter aortic valve implantation, TAVR, transcatheter aortic valve replacement, aortic valve stenosis, aortic stenosis, CT TAVI planning, transcatheter heart valve, THV

## Abstract

Transcatheter Aortic Valve Implantation/Replacement (TAVI/TAVR) has come a long way since the first-in-human implant by Prof Cribier & colleagues in 2002. Initially a consideration for inoperable/high-surgical-risk patients, TAVI is now indicated in patients with severe tricuspid aortic stenosis and suitable anatomy aged 70 or higher. This has been made possible due to improvements in preprocedural planning, performance upgrades to and evolution of transcatheter heart valve (THV) systems and increasing operator experience. Younger age at index implantation, complexities of redo TAVI planning and methods to improve THV durability are the next frontiers. This review summarizes these advancements while emphasizing preprocedural planning, current guidelines, and individualized device selection, with a brief note on polymeric heart valves—developed to overcome the disadvantageous bioprosthetic dysfunction seen with current THVs.

## 1. How It Started

The development of percutaneous aortic valves stemmed from the poor long-term results and high restenosis rates of balloon aortic valvuloplasty in high-risk inoperable patients with severe native aortic valve stenosis, culminating in the first implant by Cribier and colleagues in 2002. Initial skepticism of these devices revolved around the displaced heavily calcified in situ native valve leaflets posing coronary obstruction risk, transcatheter heart valve (THV) leaflet damage during the crimping process and perceived risk of embolization of the percutaneously placed prosthesis, in the absence of anchoring sutures.

Alain Cribier’s early work had shown that a balloon-expandable stent deployed and fixed in the native aorta could withstand up to 2 kg of tension. His work in close association with Percutaneous Valve Technologies, later acquired by Edwards in 2004, led to the initial Cribier Edwards Valve, available in sizes of 23 and 26 mm (introducer sheaths of 22 and 24F respectively). The valve was originally implanted through an antegrade transeptal approach necessitating crossing of the mitral valve, until the development of a retrograde transfemoral trans-aortic implantation technique with a deflectable pusher sheath. As an alternative and in the face of the large femoral sheaths of the time, a transapical access was initiated as a “front door approach”. However, with an almost double 30-day mortality rate, this access approach fell out of favor.

Meanwhile, on the back of successful animal implants, Eberhard Grube performed the first human implantations of the self-expanding CoreValve prosthesis in 2005—the company would subsequently be acquired by Medtronic in 2009 [1].

The initial trials—PARTNER B using a balloon-expandable valve (BEV) and CoreValve Pivotal Trial using a self-expanding valve (SEV)—showed decreased mortality at one year in inoperable severe AS patients who underwent TAVI compared to those who were on medical therapy, validating the role of TAVI as a therapeutic intervention in severe aortic stenosis [2].

A major milestone, however, was reached with the PARTNER A (2011) and CoreValve High Risk Study (2014), which showed the non-inferiority of TAVI (with a BEV and SEV respectively) with regard to all-cause mortality at 30 days and 1 year, when compared to surgical aortic valve replacement (SAVR) with a bioprosthesis, in patients at high surgical risk [2]. Risk stratification was done based on the EUROSCORE or Society of Thoracic Surgeons predicted risk of mortality score (STS score) [2].

Interestingly these trials also highlighted differences in complications between TAVI and surgical aortic valve replacement (SAVR) with vascular complications, paravalvular regurgitation (PVR) and permanent pacemaker implantation being more frequent with the former and acute kidney injury (AKI), major hemorrhage and atrial fibrillation (AF) being more common with the latter [2].

The trials that followed—PARTNER 2 and SURTAVI—involved patients with severe AS and intermediate surgical risk [3,4].

In PARTNER 2, 2032 patients with severe AS and intermediate surgical risk were randomized to undergo SAVR or TAVI using the Sapien XT prosthesis (with around 76% patients selected for transfemoral—TF—TAVI). The primary outcome was death from any cause or disabling stroke at 2 years, with TAVI found to be non-inferior to SAVR (*p* = 0.0001 for non-inferiority). Furthermore, those patients who underwent TF TAVI had lower rates of death and disabling stroke vs. those who underwent SAVR. TAVI also resulted in increased aortic valve areas (AVAs), lower rates of AKI, severe bleeding and AF, while fewer rates of paravalvular leaks (PVLs) and vascular complications were noted in the SAVR group [3].

The SURTAVI trial also involved intermediate-risk patients with severe AS with 1746 patients randomized to SAVR or TAVI (with CoreValve prosthesis used in 84% and Evolut R in 16%). TAVI was non-inferior to surgery at 2 years with regard to the primary outcome—death from any cause or disabling stroke. The pattern of complications was similar to PARTNER 2, with lower rates of AF and transfusion but higher rates of residual aortic regurgitation (AR) in the TAVI group. In addition, a higher rate of permanent pacing was found in the TAVI group [4].

Evidence of comparable efficacy in severe AS patients with low surgical risk was noted in the subsequent PARTNER 3 and Evolut Low-Risk trials [5,6].

PARTNER 3 randomized 1000 such patients to either SAVR or TF TAVI with the Sapien 3 valve. The primary endpoint, a composite of death, stroke or hospitalization at 1 year, was markedly lower in the TAVI group (8.5% vs. 15.5%; difference of −6.6 percentage points, *p* < 0.001 for non-inferiority). Patients who underwent TAVI had a shorter index hospitalization and additionally, at 30 days, TAVI also showed reduced rates of death or stroke and new-onset AF [5].

The Evolut Low-Risk trial, which randomized 1468 patients with severe AS and low surgical risk to either SAVR or TAVI with a self-expanding prosthesis (CoreValve 3.6%, Evolut R 74.1% and Evolut Pro 22.3%), observed no difference in the primary composite endpoint of death from any cause and disabling stroke at 24 months (6.7% vs. 5.3% respectively). At 30 days, the TAVI group had lower rates of disabling stroke, bleeding complications, acute kidney injury (AKI) and AF with higher rates of moderate to severe AR and permanent pacemaker implantation compared to the SAVR group. At 12 months, the TAVI group had lower gradients (8.6 vs. 11.2 mm Hg) and larger effective orifice areas (2.3 cm^2^ vs. 2.0 cm^2^) [6].

**Figure 1:**
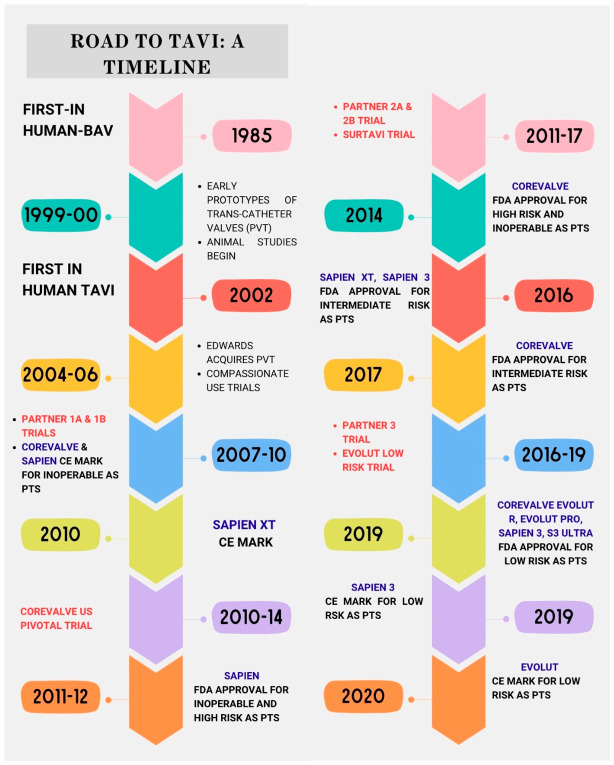
A brief timeline revealing the sequence of events from the first BAV to TAVI trials and subsequent THV approvals (major trials are in red, approved valves in blue) [7].

A timeline of these developments and landmark trials is illustrated in Figure 1.

Over time, improvements in procedural planning with computed tomography (CT), vascular access closure, valve technologies and operator experience have resulted in lower rates of procedural complications, stroke, moderate to severe AR and death [8,9,10].

### 1.1. Procedural Planning Using CT

The aortic root is an extension of the left ventricular outflow tract (LVOT) extending from the basal attachment of the aortic valve cusps to their peripheral attachment at the sinotubular junction (STJ) level. It is composed of the Sinuses of Valsalva (SoVs), the fibrous interleaflet triangles and the valve cusps themselves.

**Figure 2:**
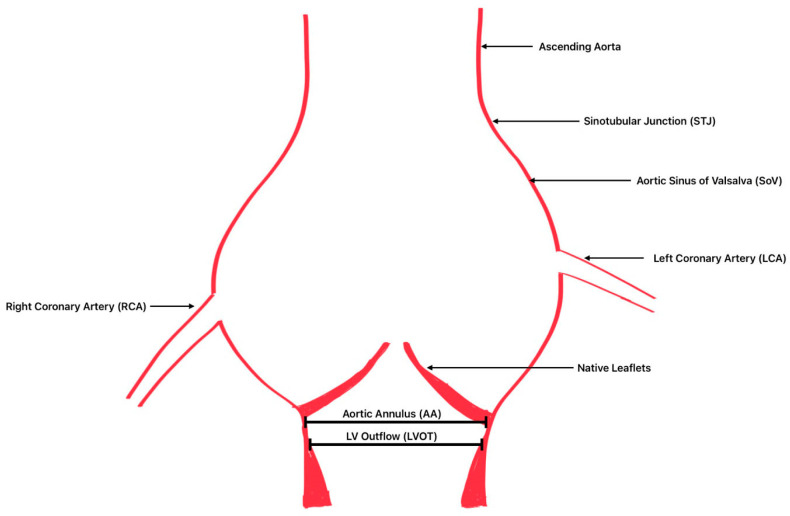
Aortic root anatomy.

The luminal contour on CT within the virtual plane that passes through the basal attachment point of each cusp represents the aortic annulus [11] (see Figure 2).

The Society of Cardiovascular Computed Tomography (SCCT) expert consensus on CT prior to TAVI published in 2012 recommended CT imaging prior to TAVI, specifically ECG-synchronized imaging of the aortic root, preferably in systole (in view of dynamic changes in annular size and slightly larger dimensions noted in systole) with additional imaging of the entire aorta from the aortic arch to below the groin, going past the common femoral bifurcation and thus enabling ileofemoral assessment. In addition to measurements of the aortic root (at annular, SoV and STJ levels), measurements needed include those of the LVOT and coronary artery heights. Interpretation jointly with a member of the TAVI team was recommended [12].

Vascular access planning on CT involves peripheral vasculature assessment to determine minimal luminal size, tortuosity and calcification to determine suitability of access and to predict probability of vascular complications. Ileofemoral arteries are assessed, and if unsuitable, other sites of vascular access (e.g., transaxillary and transcarotid) can be evaluated in this manner [8].

The updated SCCT consensus document expanded the role of CT in evaluation of the transcatheter heart valve (THV) post-TAVI, mentioning its role in assessment of suspected structural deterioration, valve thrombosis and infective endocarditis as an adjunct to echocardiography. It also described the concept of “HALT”—Hypoattenuated Leaflet Thickening—which, when seen on a post-TAVI CT scan combined with restricted leaflet motion—referred to as HAM (hypoattenuation affecting motion)—may indicate leaflet thrombus formation. Additionally, semiquantitative, subjective HALT grading on CT is possible using a long-axis multiplanar reformation (MPR) through the cusp center (<25%, 25–50%, 50–75%, >75%) [11].

Furthermore, the updated guidelines shed light on CT planning in “valve-in-valve (ViV)” (TAVI-in-SAVR) procedures, which center around defining the risk of coronary artery obstruction [11].

In recent years, “redo TAVI” (TAVI-in-TAVI/TAVI-in-THV) procedures are also becoming more common given the evolution of TAVI guidelines to include younger patients and patient survival exceeding THV durability as a consequence.

In redo TAVI, CT is fundamental in planning and allows the delineation of the “neoskirt” and “coronary risk” planes, in order to determine the risk of coronary artery obstruction [13].

While PARTNER 1 and 2 used echocardiography for measurements and sizing, in the PARTNER 3 trial, annular measurements were done on one of three 3D imaging modalities—CT, magnetic resonance imaging (MRI) and transesophageal echocardiography (TEE) [3,5,14].

The CoreValve, SURTAVI and Evolut Low-Risk trials however extensively used CT for aortic root and ileofemoral assessment [4,6,15].

### 1.2. Improvements in Vascular Access and Closure

Transfemoral access remains the option of first choice with the most experience and best results. The role of CT in assessing access site suitability cannot be overstated [3,8].

Yet vascular access site complications remain one of the most common complications encountered in TAVI, seen in as high as 14% of patients. Available data suggests greater benefit with ultrasound-guided percutaneous femoral access when compared to fluoroscopy-guided access [16].

There has however been a reduction in vascular complications and major bleeding over time, e.g., the vascular complication and major bleeding rates of 16.8 and 22.3% respectively seen in PARTNER 1B were down to 2.8 and 7.7% respectively in PARTNER 3. This is likely to be attributable in part to improving hardware with a reduction in sheath sizes from the 24F sheath used in PARTNER 1 to the smaller 14F sheath in PARTNER 3 [3,5,8,14].

Access site closure has also evolved, with ultrasound-guided vascular closure proving beneficial, allowing for visualization of the closure device and especially intraluminal conditions enabling precise closure device deployment [17].

Recent evidence has shown that a vascular closure device (VCD) strategy involving both a suture-(Prostyle^TM^/Proglide^TM^—Abbott Vascular, Santa Clara, CA, USA) and plug-based (Angioseal^®^—Terumo, Somerset, NJ, USA) method was superior to the traditional suture-based VCD strategy alone using two Prostyles/Proglides [9].

## 2. How It’s Going

The recently published outcomes of the PARTNER 3 cohorts showed that, at 7 years, among low-risk patients with symptomatic severe AS, no significant differences were observed with respect to the primary composite endpoint consisting of death, stroke and hospitalization, between those who had undergone TAVI and those who had undergone SAVR [18].

The 5-year outcomes of the Evolut Low-Risk trial similarly showed that, amongst patients with severe AS and low surgical risk who were treated with either TAVI or SAVR, rates of all-cause mortality or disabling stroke were comparable [19].

Taken together, this data indicates that, in the short and intermediate terms, hard outcomes, including all-cause mortality, are similar in low-risk severe AS patients treated with TAVI or SAVR [18,19].

Some long-term data is now becoming available. The NOTION trial, which randomized 280 patients with severe AS and low surgical risk (STS score < 4%) to either TAVI with the self-expanding CoreValve prosthesis (Medtronic^®^, Minneapolis, MN, USA) or SAVR with a bioprosthesis, found no differences in the primary composite outcome of all-cause mortality, stroke or myocardial infarction (MI) at 1 year. The published 10-year outcomes of these patients find that the original results still hold, with no significant difference in the primary composite outcome for the two groups of patients [20,21].

It is important to remember that the intermediate- and long-term-outcome trials showing parity of TAVI with SAVR involved the use of previous generations of THVs. These have since undergone further evolution and upgrades, demonstrating improved performance [18,19,20,21,22,23].

While the role of TAVI in symptomatic severe AS is established, recent evidence has shown potential benefit in asymptomatic severe AS. This is inferred from the findings of the Early TAVR trial, in which 455 patients with asymptomatic severe AS were randomized to undergo TF TAVI with a BEV or clinical surveillance, with the primary endpoint being a composite of death, stroke or unplanned hospitalization from cardiovascular causes. A primary endpoint occurred in 26.8% patients in the TAVR group vs. 45.3% patients in the clinical surveillance group (HR 0.50, *p* < 0.001) [24].

### 2.1. Current Guidelines

Symptomatic severe AS has poor prognosis without intervention. Early intervention (TAVI or SAVR) is strongly recommended in all such patients with an estimated life expectancy of more than one year [25].

A diagnosis of severe AS is established on 2D echocardiography criteria:

- Peak velocity and mean gradient across aortic valve > 4 m/s and >40 mmHg respectively with the calculated aortic valve area (AVA) being <1 cm^2^ or indexed AVA < 0.6 cm^2^/m^2^.
- In case of discordant data, other measurements indicative of flow (LV Stroke Volume Index, <35 mL/m^2^ indicative of low flow) and LV systolic function (LVEF, <50% being used as the cut-off) are noted, which guide further testing in the form of Dobutamine Stress Echocardiography (DSE) and/or Aortic Valve Calcium Score on CT (>2000 AU in males and >1200 AU in females indicative of severe AS).

The guidelines also shed light on the features that favor TAVI as a choice of intervention in these patients. These include:

Age ≥ 70 years.

Anatomic features:

○ Transfemoral access suitable for TAVI;
○ Porcelain aorta;
○ Intact coronary artery bypass grafts;
○ Severe chest deformity/scoliosis.

Concomitant conditions:

○ Comorbidities/cardiac conditions which may increase surgical risk;
○ Frailty;
○ Sequelae of chest radiation.

Finally, the decision of selecting the modality of intervention in terms of the index procedure and valve type should be made keeping in mind the lifetime management of the disease and anticipating the possibility of a repeat intervention and risks therein (Redo SAVR vs. SAVR after TAVI vs. TAVI after SAVR—ViV TAVI vs. redo TAVI) [25].

### 2.2. Basics of THV Design & Currently Available Devices

The key elements of a THV include:

1. A metallic frame;
2. Leaflets;
3. Inner skirt;
4. Outer skirt [26].

Based on the mechanism of THV deployment, THVs are classified into two groups:

○ Balloon-expandable valves (BEVs);
○ Self-expanding valves (SEVs).

Furthermore, in the SEV group, the position of the leaflets leads to a subclassification of supra-annular SEVs or intra-annular SEVs [27].

The metallic frame may be made of either a multi-phase cobalt-based alloy, or, as in the case of SEVs, nitinol (an alloy of nickel and titanium). The self-expanding nature of these valves comes from the unique properties of nitinol—super-elasticity and shape memory. Nitinol thus can be deformed and remains deformed at lower temperatures, but as ambient temperature increases, nitinol assumes its original state [26].

Leaflets can be derived from either bovine or porcine pericardial tissue. As described above, leaflet position can be intra-annular (if within the annular plane) or supra-annular. Supra-annular leaflets, not constrained by the rigid annulus, provide greater post-implant internal diameter and better hemodynamics [26].

However, supra-annular leaflet design may add complexity in case of a future redo TAVI procedure by raising the neoskirt plane (NSP) [28].

Lastly, some valves have a skirt sewn at the ad luminal surface of the metallic frame, proximal to leaflet insertion—the inner skirt. Some valves have an additional outer skirt, sewn onto the ab luminal surface of the proximal metallic frame. Skirts are made of either polyethene tetrapthalate (PET), fabric or pericardial tissue and function to reduce paravalvular leaks (PVLs) [26].

The Sapien 3 and S3 Ultra (Edwards Lifesciences^®^, Irvine, CA, USA) are trileaflet BEVs with bovine pericardium-derived leaflets mounted on a cobalt chromium frame.

The Evolut (Medtronic^®^, Minneapolis, MN, USA) is a supra-annular trileaflet SEV with leaflets derived from porcine pericardium, mounted on a nitinol frame [27].

**Figure 3:**
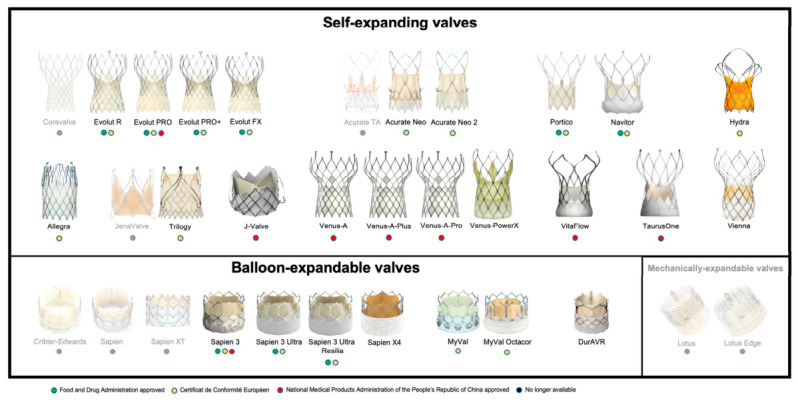
Recently available TAVR devices (picture reproduced with permission [29]).

A picture of recently available TAVI/TAVR devices is shown in Figure 3.

### 2.3. THV Selection

As described above, there is now a plethora of devices from the initial two that were available [29]. This has led to individualization of device selection, keeping in mind anatomy, patient characteristics and operator experience for optimal management and to improve outcomes [27].

Device preference by anatomy and clinical context is summarized below:
**Short-frame BEV preferred**○Coronary artery disease;○Large aortic annulus;○Horizontal aorta;○Pre-existing conduction abnormalities increasing risk of post-TAVI permanent pacing.**Tall-frame SEV preferred**Intolerance to rapid pacing;Peripheral vascular disease;Aortic annular calcification;ViV procedures;Risk of patient–prosthesis mismatch, and small aortic annuli.

These are not absolute criteria and valve selection must be individualized [27].

### 2.4. Hemodynamic Differences Between BEVs and Supra-Annular SEVs

Better hemodynamic performance of the supra-annular self-expanding THVs is well known, stemming from lower trans-valvular gradients, which also results in lower rates of patient–prosthesis mismatch [27].

In the SMART trial, patients with symptomatic severe AS and an AVA < 430 mm^2^ were randomized 1:1 to undergo TAVI with a supra-annular SEV or a BEV, with the co-primary endpoint being a composite of death, stroke, rehospitalization for HF (tested for non-inferiority) and bioprosthetic valve dysfunction (BVD, tested for superiority).

BVD was defined as a hemodynamic structural valve dysfunction with a mean AV gradient of 20 mmHg or higher, non-structural valve dysfunction defined either as severe patient–prosthesis mismatch or at least moderate AR, clinical valve thrombosis, endocarditis or aortic valve reintervention.

A total of 716 patients were treated, with the mean age being 80 years and the mean STS score being 3.3%; 87% were women. Primary composite outcome at 12 months was 9.4% in the SEV group vs. 10.6% in the BEV group (*p* < 0.001 for non-inferiority).

The AV mean gradient and mean EOAs at 12 months were 7.7 mmHg vs. 15.7 mmHg and 1.99 cm^2^ vs. 1.5 cm^2^ in the SEV and BEV groups, respectively.

More significantly, BVD was 9.4% with an SEV while it was 41.6% with a BEV; *p* < 0.001 for superiority.

The study concluded that, amongst patients with severe aortic stenosis with a small annulus (<430 mm^2^), a self-expanding supra-annular SEV was non-inferior with respect to clinical outcomes but superior with respect to bioprosthetic valve dysfunction at 12 months when compared to a BEV [30].

The recently published hemodynamic comparison between the latest-generation balloon-expandable THV—Edwards Sapien 3 Ultra Resilia (S3 UR)—and supra-annular self-expanding THV—Medtronic Evolut FX—has yielded similar results with lower mean pressure gradients and higher indexed EOAs with the Evolut FX compared to the S3 UR (7.3 (5–10.5) vs. 8.7 (7.0–10.3) mmHg, *p* < 0.001 and 1.28 (1.07–1.54) vs. 1.23 (1.04–1.44), *p* = 0.021, respectively).

It is also important to note that early safety was higher in the S3 UR group and the composite outcome of all-cause mortality and HF hospitalization at 1 year was comparable between both groups [31].

The hemodynamic advantages of the supra-annular self-expanding platform appear also to have translated to the valve-in-valve subsets, as demonstrated in the meta-analysis by Yasmin et al., where the mean trans-valvular gradient was lower in the SEV vs. BEV group [14.72 (95% CI 12.73–17.07) vs. 19.4 (95% CI 17.6–21.52) mmHg].

Maximal trans-valvular gradients at 1 year and proportion of patients with severe patient–prosthesis mismatch were also lower in those receiving an SEV.

No significant differences in all-cause mortality were noted between the two groups at 30 days, 1 year and 3 years [32].

It is important to note that despite the proven better hemodynamic performance with the supra-annular self-expanding platform, evidence of its translation into unequivocally positive hard clinical outcomes, in comparison to BEVs, is still awaited, at least in native aortic valve disease, with some previous studies in fact showing the opposite.

In a French longitudinal cohort study, information for all consecutive patients treated with a TAVI device, commercialized in France between 2014 and 2018, was collected and propensity score matching was done to analyze the outcomes during follow up of TAVI with the Sapien 3 BEV and Evolut R SEV.

After matching, 20,198 patients were analyzed, 10,459 in each group. During follow up (mean 358 days, median 232 days), BEV TAVI was associated with a lower yearly incidence of all-cause death (RR 0.88; corrected *p*—0.005), cardiovascular death (RR 0.82; corrected *p*—0.002) and rehospitalization for HF (RR 0.84; *p* < 0.0001).

BEV TAVI was also associated with lower rates of permanent pacemaker implantation after the procedure (RR 0.72; *p* < 0.0001).

The study concluded that the Sapien BEV was associated with lower rates of all-cause mortality, cardiovascular death, rehospitalization for HF and permanent pacemaker implantation after TAVI [33].

The supra-annular position of the leaflets postulated to give better hemodynamics may be disadvantageous when being considered in a relatively younger patient likely to outlive their first THV—raising the neoskirt plane height potentially increases coronary obstruction risk in a redo TAVI scenario [28].

Interestingly, the Navitor THV (Abbott^®^, Santa Clara, CA, USA), an intra-annular self-expanding THV, has shown comparable hemodynamic performance and safety outcomes to the Medtronic Evolut, a supra-annular self-expanding THV. Large-scale studies however are needed to further strengthen this conclusion [34].

### 2.5. Coronary Artery Disease (CAD) & AS: Timing of Coronary Intervention with Respect to TAVI

Patients with AS and CAD undergoing SAVR have concomitant coronary artery bypass grafting (CABG) for CAD treatment.

Around half the patients undergoing TAVI have concomitant CAD and a third of these have multivessel involvement and elevated SYNTAX scores [35].

Therefore, clarifying the need for and understanding the feasibility of interventional management for CAD becomes important.

It is now increasingly realized that tall-frame THVs with supra-annular leaflets present a greater challenge for selective coronary engagement.

Therefore, alignment of THV commissures with the native aortic valve commissures—“COMMISSURAL ALIGNMENT”—becomes necessary to avoid a THV commissural post aligned across the coronary ostium and precluding coronary access. Commissural alignment should always be preferred when implanting a tall-frame THV to facilitate coronary access after TAVI, if needed.

Commissural alignment also has implications in redo TAVI planning. Specifically, commissural alignment (between the index THV and native aortic valve) is essential when considering leaflet cutting/modification techniques such as BASILICA to overcome the risk of coronary obstruction (NSP higher than CRP and lower/unfavorable VTA distances).

Commissural alignment however may not be the right parameter in cases of the coronary arising eccentrically (rather than from the center of the cusp) and in cases of bicuspid aortic valves. Here “CORONARY ALIGNMENT” with the index THV must be considered [28,36].

The EAPCI consensus statement recommends considering the possibility of coronary access during pre-TAVI CT evaluation, in patients with a history of CAD or those with CAD that may require future intervention [36].

The PRO TAVI trial, an open-label randomized controlled trial, sought to answer the question of timing of PCI in patients with CAD and AS undergoing TAVI—specifically whether the deferral of PCI was non-inferior to routine PCI in patients with AS and CAD undergoing TAVI.

CAD was defined as a stenosis of 70–99% or at least one stenosis between 40 and 70% combined with a positive physiological measurement in a coronary artery with a minimum diameter >2.5 mm. Stratification after randomization was done based on the presence of CAD involving the proximal LAD artery. The primary endpoint was all-cause mortality, stroke or VARC3 Type 2–4 bleeding at 12 months after randomization. Important exclusion criteria included CAD involving unprotected left main (LM) disease/LM equivalent disease and non-PCI-eligible stenoses.

Out of 466 patients enrolled, 233 were assigned to the deferral group and 233 to the PCI group. Both groups had similar baseline characteristics with a baseline SYNTAX score of 10. The type of THV to be used was left to the heart team to decide. The median time to TAVI from randomization in the deferral group was 25 days while in the PCI group it was 31 days (with the median time to PCI being 13 days).

The primary endpoint occurred in 24% patients in the deferral group vs. 26% in the PCI group, with the rate difference of −1.7% meeting the criteria for non-inferiority but not superiority. Major bleeding was more frequent in the PCI-first group (15% vs. 6%).

In the deferral group, 24 out of the 233 patients underwent PCI with a median time of 87 days between randomization and the procedure, with 13 patients needing urgent revascularization and 11 elective revascularization (9 due to symptoms and 2 due to treating physician preference). Post-TAVI PCI was only done for persistent complaints of anginal pain or ischemic signs.

At the one-year follow up, only one patient in the deferral group had angina CCS III symptoms.

The trial concluded that in patients with CAD undergoing TAVI, deferral of PCI was safe and non-inferior to a pre-TAVI PCI approach [37].

However, there is a need for a nuanced approach when attempting to answer the question of timing of PCI in patients with AS and CAD undergoing TAVI, partly influenced by the indication of PCI.

While the role of PCI in acute coronary syndromes (ACSs) is well established, its role in chronic coronary syndromes is less so, where there is equipoise with optimal medical therapy in terms of survival [38,39].

Patients with significant CAD with LM involvement/triple-vessel disease, especially with LV dysfunction, are known to have improved outcomes with CABG over medical therapy alone [39], but this group of patients was excluded from the PRO TAVI trial [37].

## 3. Where It’s Going

Indications for which TAVI has been considered, beyond severe tricuspid aortic stenosis, include:

○ Severe bicuspid aortic valve stenosis;
○ Severe native aortic valve regurgitation;
○ Moderate aortic stenosis.

###  

#### 3.1.1. Bicuspid Aortic Valve Disease

Bicuspid aortic valve—BAV—is a congenitally deformed aortic valve where the valve closure mechanism is marked by less than three distinct and parallel areas of apposition between valve cusps, sometimes marked by the presence of a “raphe”.

The “Raphe”, a ridge composed of elastic fibers, is a malformed commissure representing the fusion zone of two adjacent, underdeveloped cusps. In contrast, an actual “commissure” of the aortic valve represents the space between the parallel, lateral cuspal leaflet attachments to the aortic wall.

These terminologies require clarification because the spectrum of BAV extends from the complete absence of one commissure resulting in two cusps, sinuses and commissures and a “pure” bicuspid anatomy, to partial development of one or two commissures and adjacent leaflet cusps, resulting in the more commonly encountered variety of bicuspid aortic valve anatomy, with one or two raphes.

**Figure 4:**
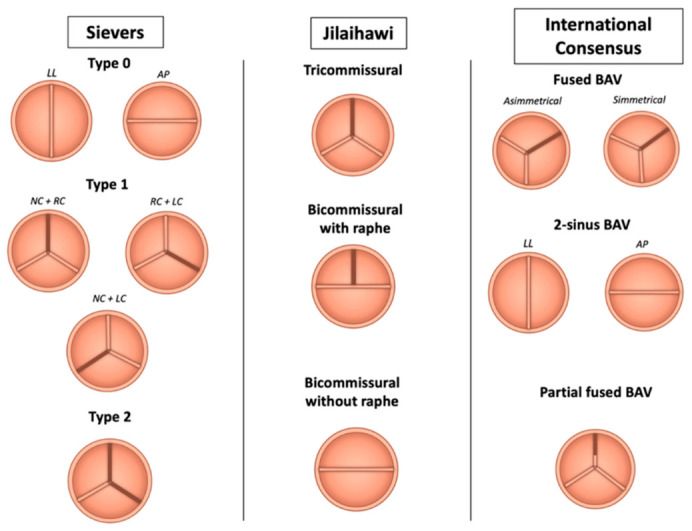
Anatomical classification of bicuspid aortic valve phenotypes. Abbreviations: AP = antero-posterior; BAV = bicuspid aortic valve; LC = left cusp; LL = latero-lateral; NC = non-coronary cusp; RC = right cusp (picture reproduced with permission [40]).

The Sievers classification system, which sought to bring greater simplicity and accuracy to the discourse around BAV, is based on the number of raphes—the most important point (for which “Type” is accorded), followed by the orientation of cusps and raphe(s) and the hemodynamic, functional status of the valve (see Figure 4).

In the retrospective study by Sievers et al., Type 1 BAV with L-R fusion was the most commonly noted type of bicuspid anatomy, whilst aortopathy with ascending aortic aneurysms was noted significantly in Type 2 BAV (BAV with two raphes) [41].

Bicuspid aortic valve disease is manifested by early valve deterioration with patients presenting with severe stenosis at a younger age and with associated aortopathy in up to half of these individuals. Decisions on utilization of TAVI in this group therefore need to keep in mind lifetime management of the disease, with a potential redo procedure needed in the future [42].

TAVI in BAV disease presents several technical challenges, both at the stages of preprocedural planning and during the procedure itself. THV sizing in TAVI is based on the virtual aortic annular plane, passing through the hinge points of all three aortic valve cusps, with this being the tightest portion of the aortic root. In bicuspid anatomy however, owing to asymmetric cusps and extensive eccentric calcifications, including those of the raphe when present and an elliptical annular plane, identification of the most limiting dimension of the aortic root, for the purposes of determining appropriate THV sizing, is not straightforward. Furthermore, due consideration must also be given to the presence of a raphe, its calcification and calcification of leaflets—the presence of both of which is associated with a four-fold increase in mortality at 2 years.

Additionally, it has been shown that Type 0 BAV patients undergoing TAVI have a better long-term prognosis compared to Type 1 BAV patients—owing to the asymmetric THV expansion in the latter group due to the presence of a raphe.

Multiple CT sizing methods have been developed for AS patients with bicuspid valves, each with its own advantages and disadvantages, in an attempt to achieve the best possible sizing and hemodynamic success, while mitigating the risk of PVL and aortic root injury. The BAVARD method for example uses an intercommissural diameter at 4 mm above annulus (ICD 4) to determine a supra-annular taper (if <annular diameter) vs. tubular or flare (≥annular diameter), with a recommendation to size per ICD in a tapered configuration. The more intuitive CIRCLE method uses a device-sized CIRCLE traced across when scrolling through the root at 3 mm intervals, with the first full-contact circle defining the size. A detailed discussion of these methods is beyond the scope of this review.

Procedural challenges are centered around valve crossing and navigating horizontal aortic roots/tortuous aortic anatomy. Using delivery systems with flexibility (BEV devices for example) or a snare or a “buddy-balloon” maneuver is useful in overcoming these challenges. Post-dilatation must be considered for clear indications of raised trans-valvular gradients or significant PVL and must be balanced against the risk of aortic injury [40].

TAVI in bicuspid valve disease was initially limited to inoperable or high-surgical-risk patients with severe bicuspid aortic stenosis. Additionally, trials that compared TAVI with SAVR largely excluded patients with bicuspid aortic valve disease, with the exception of the UK TAVI and Notion 2 trials.

In the NOTION 2 trial, the composite endpoint (all-cause mortality, stroke or procedure/valve/heart failure) was higher in the subgroup of patients with bicuspid aortic valve disease (and low surgical risk) who underwent TAVI (49 patients) compared to those who underwent SAVR (51 patients) at 3 years. While these results did not achieve statistical significance, this has to be understood in the context of the trial being insufficiently powered to gain a meaningful result in this subgroup.

Current guidelines recommend SAVR as the treatment of choice in patients with bicuspid aortic valve disease. However, TAVI is superior to medical therapy alone in high-risk inoperable patients with severe bicuspid AS.

Two upcoming trials, NAVIGATE Bicuspid and BELIEVERS, will directly compare TAVI to SAVR in patients with bicuspid aortic valve disease [42].

#### 3.1.2. Severe Aortic Valve Regurgitation

TAVI in severe native aortic regurgitation may be considered if the patient is ineligible for surgery per the heart team and if their anatomy is suitable (IIb) [25].

The ALIGN AR was a prospective, multi-center, single-arm study that looked at the effectiveness of treating native moderate to severe aortic regurgitation, in patients deemed to be at high surgical risk by the heart team, with the Trilogy transcatheter heart valve (JenaValve Technologies^®^, Irvine, CA, USA). The 30-day primary safety composite endpoint of 25% achieved non-inferiority, well below the expected prospective performance goal of 40.5%. This demonstrated the safety and effectiveness of treating severe native AR using a dedicated THV system [43].

#### 3.1.3. Moderate Aortic Valve Stenosis

At the present time, there is not enough evidence to prove efficacy of TAVI in patients with moderate AS, with surgical intervention recommended only if the patient is undergoing concomitant CABG [25].

The TAVR UNLOAD trial randomized 178 patients with moderate AS (AVA ≤ 1.5 cm^2^, iAVA < 1.0 cm^2^ or not meeting criteria for severe AS on DSE) and with HFrEF (LVEF 20–50%, NYHA II–IV, receiving appropriate and stable GDMT for at least 1 month) to TAVI with a BEV vs clinical surveillance. While TAVI resulted in a greater improvement in the KCCQ overall summary score, it was deemed to be non-superior to clinical surveillance with regard to the primary endpoint—a hierarchical occurrence of all-cause death, disabling stroke, disease-related hospitalizations and HF equivalents and change in the baseline KCCQ overall summary score [44].

### 3.2. Bioprosthetic Valve Dysfunction and Improving THV Durability as Part of Lifetime Management

While current guidelines recommend consideration of TAVI in patients with severe aortic stenosis and suitable anatomy aged 70 years and above, TAVI use has skyrocketed in patients less than 65 years of age, with implantation rates increasing by 8 times—from 7.1% in 2013 to 54.7% in 2021 [25,45].

Increasing numbers of younger patients undergoing TAVI coupled with increasing patient survival is likely to result in more patients outliving their first THV, the downstream effects of which include redo TAVI planning and its associated complexities [13,28].

It is paramount, therefore, to understand mechanisms of THV (bioprosthetic) dysfunction and durability and performance of THVs beyond a 10–15-year period.

Bioprosthetic valve dysfunction, which can be subdivided into structural vs. non-structural dysfunction, occurs due to one of the following pathologic mechanisms:

○ Pannus formation;
○ Paravalvular regurgitation;
○ Patient–prosthesis mismatch;
○ Endocarditis or thrombosis.

Subclinical leaflet thrombosis (SLT), defined as presence of leaflet thickening with reduced motion (on echocardiography or CT) without hemodynamic dysfunction/cardiac symptoms or TIA (VARC 3), has been reported in as high as 30% of post-TAVI patients at 1 year.

SLT may escalate toward clinically significant thrombosis, which has been shown in meta-analysis to be associated with a three-times-increased risk of stroke.

Despite this, systematic use of oral anticoagulants—OACs—has not been found to be useful after TAVI. Trials done in this regard did show a reduction in SLT; however this was counterbalanced by an increased risk of major bleeding complications and death [45].

Recent efforts have focused on development of TAVI valves with novel materials that enhance durability. The bioprosthetic valve dysfunction seen with current THVs has led to the demand to create non-thrombogenic durable polymeric heart valves (PHVs). For this purpose, poly (carbonate urea) urethanes (PCUs) have been found to be a viable class of polymeric compounds. Leaflets made from these materials resist oxidative and hydrolytic degradation. Additionally, it is postulated that they create a non-thrombotic environment by polymer endothelialization.

The Foldax Tria valve is one of the first PHVs to be tested in humans, although via surgical implantation. At 1-year follow up, out of the 14 patients who underwent implantation, 2 patients died due to valve-related causes. However, hemodynamic performance and NYHA class were maintained throughout the follow-up period [45].

A study published in April 2026 demonstrated the safety and efficacy of TAVI with the Sikelia transcatheter heart valve—a self-expanding valve featuring leaflets made from polyurethane materials. At discharge, out of the 12 patients who underwent TAVI, there were no reported deaths, strokes or requirements for permanent pacemaker implantation. At 1 year the mean pressure gradient improved substantially (6.33 ±2.54 mmHg vs. 47.5 ±13.82 mmHg). No moderate or severe PVL and no evidence of SLT were noted on CT during follow up. While a favorable hemodynamic performance and acceptable safety profile was noted at 1 year, long-term follow up would be needed to assess durability before consideration of this technology as a treatment for patients with severe AS [46].

### 3.3. Cerebral Embolic Protection During TAVI Procedures

Multiple trials have studied the role of cerebral embolic protection in TAVI, with no significant benefit noted.

In the multinational PROTECTED TAVR trial which used the Sentinel (Boston Scientific^®^, Marlborough, MA, USA) cerebral embolic protection (CEP) device, 3000 patients were randomized either to TAVI with CEP or TAVI without the use of embolic protection (control group). The primary outcome of stroke within 72 h of the procedure or before discharge did not differ significantly between both groups. Disabling strokes were lower in the CEP group (0.5 vs. 1.3%, −0.8 difference; 95% CI −1.5 to −0.1), but the trial was not adequately powered for assessing disabling stroke [47].

The subsequent UK-based multi-center BHF PROTECT TAVI trial enrolled 7635 patients with severe aortic stenosis, randomized 1:1 to TAVI with a CEP device or without while looking primarily at stroke within 72 h after TAVI or before discharge. Despite less restrictive enrollment criteria and the requirement of having both device filters fully deployed for the entirety of the procedure, there were no significant differences with regard to the primary outcome between the two groups, in line with the results of the PROTECTED TAVR trial.

In addition, no significant differences were noted with regard to disabling stroke rates [48]. Two recent meta-analyses have concluded that cerebral embolic protection during TAVI neither significantly reduced stroke incidence nor reduced all-cause mortality [49,50].

### 3.4. Pacemaker Risk Post-TAVI: Where Are We Now?

The risk of permanent pacing after TAVI, while common to both BEV and SEV [51,52], is considered greater with the use of an SEV, with pacemaker implantation being associated with increased costs, extended hospitalization and potentially adverse patient outcomes [6,33,53].

While pre-existing conduction abnormalities, especially a right bundle branch block (increases the risk five-fold), and depth of THV implantation were common predictors of the need for permanent pacing between both BEV and SEV, there were some differences.

For example, in BEV, a depth of implantation of more than 6 mm almost doubled the risk of permanent pacing, whereas for an SEV this cut-off was at 3 mm.

Additionally, the use of a larger prosthesis and the presence of annular calcium were additional risk factors that doubled the risk of requiring pacing in the BEV group [51,52].

The two-cusp-overlap angiographic projection, brought about by overlapping the right and left coronary cusps to one side and isolating the lowermost non-coronary cusp on the other, was hypothesized to allow for better depth assessment without LVOT foreshortening, both of which were disadvantages of the three-cusp coplanar projection.

The use of this projection for device implantation has resulted in a reduction in permanent pacemaker implantation rates post-TAVI when used with SEVs and has thus become a critical angiographic projection during SEV deployment [54,55].

## 4. Conclusions

TAVI has come a long way from the initial implantations done in inoperable patients, aided by improvements in preprocedural planning (CT), THV technologies and operator experience.

Current indications now extend to patients with symptomatic severe tricuspid aortic stenosis aged 70 years or more whilst use in aortic regurgitation and bicuspid aortic valve disease requires further evidence.

The current paradigm involves valve selection, from the available BEVs and SEVs, individualized to the patient’s unique anatomical features on CT.

Lower ages at initial implantation have resulted in an emphasis on redo TAVI planning and methods to improve THV durability. Polymeric heart valves (PHVs) developed for this purpose appear promising, although larger trials and longer term assessment are needed.

## Data Availability

The original contributions presented in this study are included in the article Further inquiries can be directed to the corresponding authors.
